# A Newfangled Collagenase Inhibitor Topical Formulation Based on Ethosomes with *Sambucus nigra* L. Extract

**DOI:** 10.3390/ph14050467

**Published:** 2021-05-15

**Authors:** Ana Henriques Mota, Inês Prazeres, Henrique Mestre, Andreia Bento-Silva, Maria João Rodrigues, Noélia Duarte, Ana Teresa Serra, Maria Rosário Bronze, Patrícia Rijo, Maria Manuela Gaspar, Ana Silveira Viana, Lia Ascensão, Pedro Pinto, Pradeep Kumar, António José Almeida, Catarina Pinto Reis

**Affiliations:** 1 iMED.Ulisboa, Research Institute for Medicines, Faculdade de Farmácia, Universidade de Lisboa, Av. Prof. Gama Pinto, 1649-003 Lisboa, Portugal; ana.luisa.mota@campus.ul.pt (A.H.M.); henriquemestre@campus.ul.pt (H.M.); abentosilva@ff.ulisboa.pt (A.B.-S.); mduarte@ff.ulisboa.pt (N.D.); mrbronze@ff.ulisboa.pt (M.R.B.); patricia.rijo@ulusofona.pt (P.R.); mgaspar@ff.ulisboa.pt (M.M.G.); aalmeida@ff.ulisboa.pt (A.J.A.); 2 IBET, Instituto de Biologia Experimental e Tecnológica, Av. da República, Estação Agronómica, Apartado 12, 2780-901 Oeiras, Portugal; ines.prazeres@ibet.pt (I.P.); tserra@ibet.pt (A.T.S.); 3 Instituto de Tecnologia Química e Biológica António Xavier, Universidade Nova de Lisboa (ITQB NOVA), Av. da República, 2780-157 Oeiras, Portugal; 4 Centre of Marine Sciences, Faculty of Sciences and Technology, University of Algarve, Ed. 7, Campus of Gambelas, 8005-139 Faro, Portugal; mary_p@sapo.pt; 5 CBiOS—Research Center for Biosciences & Health Technologies, Universidade Lusófona de Humanidades e Tecnologias, Campo Grande 376, 1749-024 Lisboa, Portugal; 6 Centro de Química Estrutural, Faculdade de Ciências, Universidade de Lisboa, Campo Grande, 1749-016 Lisboa, Portugal; apsemedo@fc.ul.pt; 7 Centro de Estudos do Ambiente e do Mar (CESAM), Faculdade de Ciências, Universidade de Lisboa, Campo Grande, 1749-016 Lisboa, Portugal; lmpsousa@fc.ul.pt; 8 PhDTrials—International Contract Research Organization, Av. Maria Helena Vieira da Silva nº 24 A, 1750-182 Lisboa, Portugal; pcontreiras@sapo.pt; 9 Wits Advanced Drug Delivery Platform Research Unit, Department of Pharmacy and Pharmacology, School of Therapeutic Sciences, Faculty of Health Sciences, University of the Witwatersrand, Johannesburg 2193, South Africa; pradeep.kumar@wits.ac.za; 10 IBEB, Biophysics and Biomedical Engineering, Faculdade de Ciências, Universidade de Lisboa, 1749-016 Lisboa, Portugal

**Keywords:** *Sambucus nigra* L., ethosomes, collagenase inhibition, skin compatibility

## Abstract

*Sambucus nigra* L. (*S. nigra*) is a shrub widespread in Europe and western Asia, traditionally used in medicine, that has become popular in recent years as a potential source of a wide range of interesting bioactive compounds. The aim of the present work was to develop a topical *S. nigra* extract formulation based on ethosomes and thus to support its health claims with scientific evidence. *S. nigra* extract was prepared by an ultrasound-assisted method and then included in ethosomes. The ethosomes were analyzed in terms of their size, stability over time, morphology, entrapment capacity (EC), extract release profile, stability over time and several biological activities. The prepared ethosomes were indicated to be well defined, presenting sizes around 600 nm. The extract entrapment capacity in ethosomes was 73.9 ± 24.8%, with an interesting slow extract release profile over 24 h. The extract-loaded ethosomes presented collagenase inhibition activity and a very good skin compatibility after human application. This study demonstrates the potential use of *S. nigra* extract incorporated in ethosomes as a potential cosmeceutical ingredient and on further studies should be performed to better understand the impact of *S. nigra* compounds on skin care over the time.

## 1. Introduction

The skin constitutes the interface between the organism and the external environment, acting as an epidermal barrier, protecting and supporting the life that it encloses. It presents three different layers (epidermis, dermis and hypodermis). The epidermis in turn can be divided in distinct layers: stratum corneum, lucidum, granulosum, spinosum and basale [1,2,3].

Over the years, skin disorders have been emerging, affecting millions of people particularly in developing countries. Several factors can disturb this barrier function by damaging the stratum corneum (SC). These factors are associated with ultrastructural anomalies in the upper granular layer [1], related to a change of integrity of the skin layers and respective structures. These disorders can appear due to many factors, underlying medical conditions and other pathologies, such as inflamed skin, infections, chronic inflammatory skin disorders (e.g., psoriasis), sensitive skin (e.g., allergic contact dermatitis, atopic dermatitis, seborrhea dermatitis or rosacea), among others [1]. Inflamed skin is generally characterized by redness, swelling, itching, heat and pain [2]. The cause or trigger for skin inflammation may be acute or chronic. Most cases are curable, and the treatment depends on what is causing the inflammation. On the other hand, skin infections occur when bacteria, virus, fungus or other foreign substances enter the skin through a wound or cut. These can lead to several diseases like cellulitis, impetigo, staphylococcal infections, shingles, warts, yeast infections, among others. Symptoms include swelling, redness, pain, irritation, itching and burning sensation. Finally, sensitive skin is described as a discomfort situation with stinging, burning or tingling sensations and sometimes it can be very painful and itch [2]. Thus, finding new protective bioactive ingredients that can help in skin care is crucial. 

*S. nigra* has been used in traditional medicine for several purposes [4,5]. One of them is related to its microbiological properties. Recent studies reported that a flower extract of *S. nigra* has several important biological activities, such as anti-inflammatory properties and collagenase (Coll)-inhibitory activity [4,5]. Coll is a matrix metalloprotease (MMP) enzyme responsible for the degradation of collagen [5,6]. In general, this enzyme is produced by fibroblasts like synoviocytes [6], and the degradation of the extracellular matrix by Coll has an important part in the invasion of tumor cells [7]. Collagen can be classified in different types, being collagen type I, the most abundant protein of the skin [8] and collagen I and III fibrils, responsible for the strength and resiliency of the skin. The skin aging is associated with an overexpression of Coll activity [9], as well as a reduced production of type I and III collagen as well as an abundance of degraded and disorganized collagen fibrils [8,10]. Furthermore, tumor cells may induce host cells to produce Coll [7]. In this sense, like many other species, elderflower extracts might have some potential and interesting properties against skin aging, although, its direct skin application can be compromised by thermal degradation [11], or by photodegradation which might affect its phenolic content [12]. Thus, the encapsulation or entrapment of extracts using protective carriers may present several advantages [4,13]. The characterization of the extract was already performed by our research group [4,5]. It was possible to identify malic acid, rutin, isoquercetin, isorhamnetin-3-*O*-glucoside, naringenin, quercetin-4-*O*-glucoside, isorhamnetin-3-rutinoside and luteolin-7-*O*-glucoside (see Supplementary Material Figure S1).

Nanotechnology has been used in the delivery of therapeutic agents or active ingredients through the skin. Among nanocarriers, vesicular systems are frequently used, since they present many advantages as enhancer agents of the stability of loaded agents, preventing its degradation and improving its penetration across the skin. These advantages are a consequence of their size, elasticity and lipid content, which allows to interact with the SC [2]. Among vesicles, ethosomes represent the third generation of elastic lipid carriers. They are phospholipid nanovesicles with ability to overcome the natural dermal barrier and capacity to delivery drugs through the skin layers. Ethosomes are constituted by phospholipids (20–45%), water and high concentrations of short chain alcohols (in general between 20–45% ethanol) and isopropyl alcohol or propylene glycol (up to 15%). A contributing factor that could explain the enhanced delivery when compared with other vesicles, such as liposomes, is the presence of ethanol, a known permeation enhancer, which provides ultradeformability. Ethanol is interspersed in the intercellular lipids, enhancing the lipid fluidity and decreasing the density of the lipid multilayer, event that is known as “ethanol effect”. Afterwards, the “ethosomes effect” occurs, consisting of the interlipid penetration and permeation by the opening of new pathways due to the malleability and fusion of these nanovesicles with skin lipids. Thus resulting in the release of bioactive compounds into the deep layers of the skin [14]. 

This study aimed to develop a possible ultradeformable skin carrier for *Sambucus* extract, to protect the bioactive compounds from degradation and to obtain a slow bioactive compound release [1,2,3,15,16]. 

## 2. Results and Discussion

### 2.1. Physical Characterization of Ethosomes 

**Figure 1:**
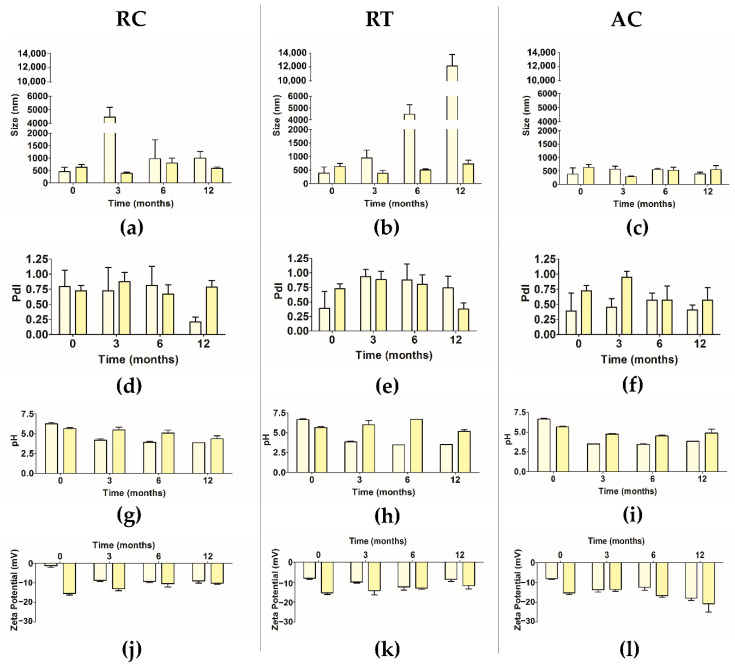
(**a**–**l**) Variation of mean size, PdI, pH and zeta potential over the time (0, 3, 6 and 12 months) of empty ethosomes (light yellow columns) and extract-loaded ethosomes (dark yellow columns) using different temperature of storage, namely, RC, RT and AC (mean ± S.D., *n* = 9).

Empty and extract-loaded ethosomes were successfully produced and then characterized in terms of size, polidispersity index (PdI) and pH. This characterization was also performed for stored particles at three different temperatures: refrigerated conditions (RC), room temperature (RT) and accelerated conditions (AC) for 12 months as depicted in Figure 1.

It is noteworthy that an increase of the size was observed after the extract encapsulation. The temperature of storage had influence on particle size. After loading the particle size of extract-loaded ethosomes was 630.1 ± 113.8 nm at RC (0 months) and 573.3 ± 192.3 nm (0 months) at AC, but a slight decrease was observed after 12 months in storage. The final particle size was 580.7 ± 57.1 nm and 549.8 ± 147.4 nm for RC and AC, respectively. However, at RT, the extract-loaded ethosomes presented an increase of the particle size from 601.6 ± 103.6 nm to 726.4 ± 133.6 nm (12 months). But, in all cases, those changes were very small in particular at AC. The particle size has influence in many properties of particulate materials, being a crucial key factor for quality and performance of the formulation [4,17]. It is very important to maintain this parameter over the time and to control the tendency of creating agglomerates. 

In terms of the PdI, at RC, the extract-loaded ethosomes presented a slight increase of PdI from 0.725 ± 0.086 (0 months) to 0.784 ± 0.111 (12 months). While, at RT and AC there was a reduction of the PdI, from 0.731 ± 0.116 (0 months) to 0.707 ± 0.194 (12 months) and 0.755 ± 0.111 (0 months) to 0.569 ± 0.210 (12 months), respectively. 

In terms of pH, after the extract encapsulation, a decrease of the pH was verified. Concerning the pH values, the extract-loaded ethosomes suspension revealed differences (*p* < 0.001) over time at RC and at AC. Less evident differences were observed at RT with a *p* < 0.01 (0–12 months). This data is in accordance with previous studies where the pH values were 4.11 ± 0.04 and 3.90 ± 0.03, for empty and rutin-loaded ethosomes, respectively [18].

Finally, the zeta potential analysis showed that the empty and extract-loaded ethosomes presented negative charge. When comparing both, i.e., empty and loaded ethosomes, the results suggest that the extract encapsulation led to an increase of the negative charge of the ethosomes.

As section summary, the extract-loaded ethosomes were stable over time in terms of size and pH. The temperature of storage had influence on the tested parameters especially on PdI. The extract-loaded ethosomes showed a better stability at RT in terms of size, PdI, pH and zeta potential when compared with other temperatures. 

### 2.2. Lipid Quantification of Ethosomes

Considering the nature of these carriers, lipid composition was determined to correctly assess the amount of ethosomes for the next studies. The calibration curve was established between 10 and 80 nmol/tube (*y* = 0.013*x* + 0.0033 with a *R*² of 0.9996). The theoretical concentration for this formulation was of 25.95 µmol/mL. However, the obtained result suggested a concentration of 28.33 µmol/mL. Thus, the result was near the empiric concentration. 

### 2.3. Histochemical Characterization of Ethosomes Lipidic Constitution and of Their Morphology 

**Figure 2:**
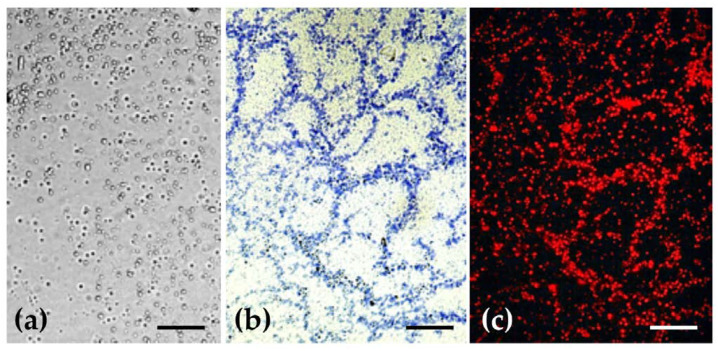
Light micrographs of empty ethosomes. (**a**) Unstained and observed with Nomarsky optics; (**b**) Stained with Nile blue A and observed under bright field illumination; (**c**) Stained with Nile blue A and observed under green light. Note the bright red secondary fluorescence of lipids. Scale bars = 5 μm.

In light microscopy, the empty ethosomes in suspension state had the appearance of small spherical granules (Figure 2a). After staining with Nile blue A (a stain for in situ detection of all the major classes of cellular lipids), they stained blue, indicating the presence of acidic lipids (Figure 2b). Neutral lipids generally stain red with this histochemical test. When Nile Blue A was used as a fluorochrome, an intense bright red fluorescence was observed under green light (Figure 2c) which confirms the lipidic constitution of ethosomes.

The micrographs obtained with Nile blue support the quantification of lipids determined previously, where the same tendency was observed. In Figure 2c, the amount of red points was significant, revealing an important amount of lipids into the formulation, which corresponds to the phosphatidylcholine (SPC) used. 

**Figure 3:**
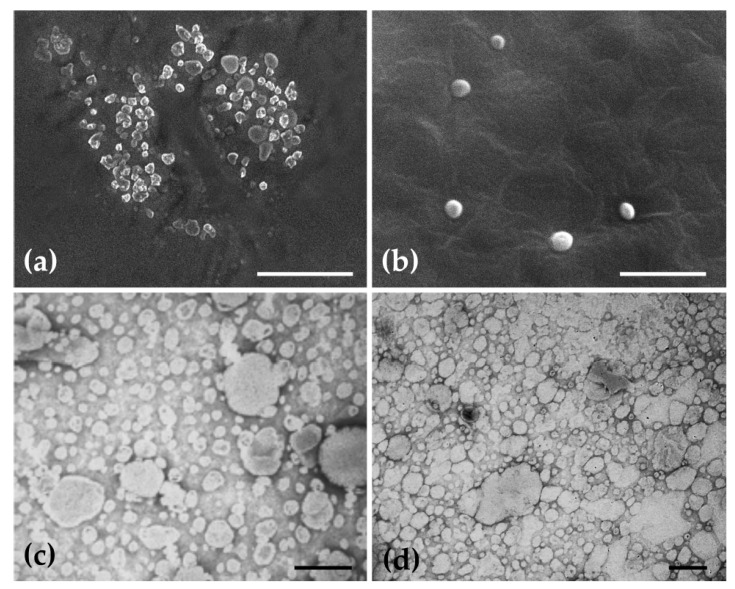
Representative SEM and TEM micrographs showing the morphology of ethosomes. (**a**,**b**) SEM images of empty and extract-loaded ethosomes, respectively. (**c**,**d**), TEM images of empty and extract-loaded ethosomes, respectively. Scale bars = 5 µm (**a**); 3 µm (**b**); 2 µm (**c**,**d**).

**Figure 4:**
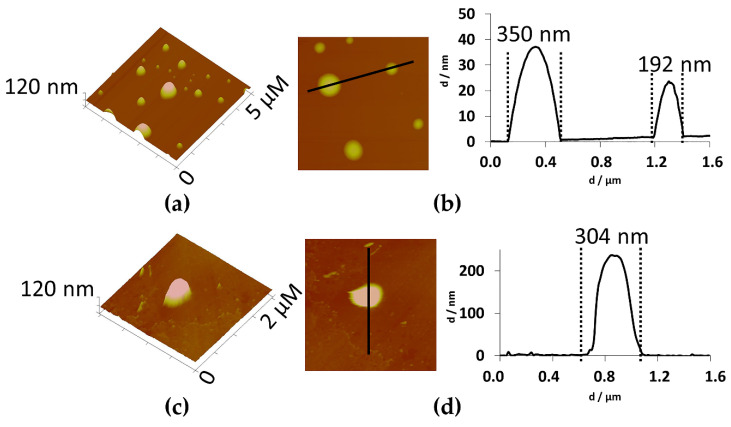
AFM topographic images of ethosomes. (**a**,**b**) empty and (**c**,**d**) extract-loaded. 3D images (**a**,**c**) and cross section analysis (**b**,**d**).

Furthermore, empty and extract-loaded ethosomes were observed by scanning and transmission electron microscopy (SEM and TEM), as well as by atomic force microscopy (AFM). By SEM, all ethosomes presented a spherical or a very near-spherical-shape and a smooth surface (Figure 3a,b). The morphology of ethosomes does not seem to change after entrapment of the extract. TEM observations also showed spherical shaped unilamellar vesicles (Figure 3c,d), confirming the results obtained by Dynamic Light Scattering (DLS). By AFM (Figure 4), another technique and sample treatment, sizes between ca 140 and 350 nm were observed for empty ethosomes, whereas extract-loaded ones exhibited approximately 300 nm, as represented in the cross-section profiles of Figure 4b,d. Overall, the sizes observed by AFM were lower than the ones determined by DLS. AFM generally delivers a high contrast images and signal to noise ratio. However, it can be very sensitive to cleanliness of the samples [19]. In case of DLS, this technique measures Brownian motion and relates to the hydrodynamic diameter. This value refers to how a particle diffused within a fluid and it strongly depends on the particle “core”, particle surface as well the concentration and type of the ions in that medium that forms a double layer. These ions can influence the thickness of the electric double layer. All techniques showed that empty and extract-loaded ethosomes exhibited a spherical or near-spherical-shape, which is a good indicator for the potential topical use, since the surface area versus volume should be high in principle [20]. 

### 2.4. Entrapment Capacity of Extract in Ethosomes

**Figure 5:**
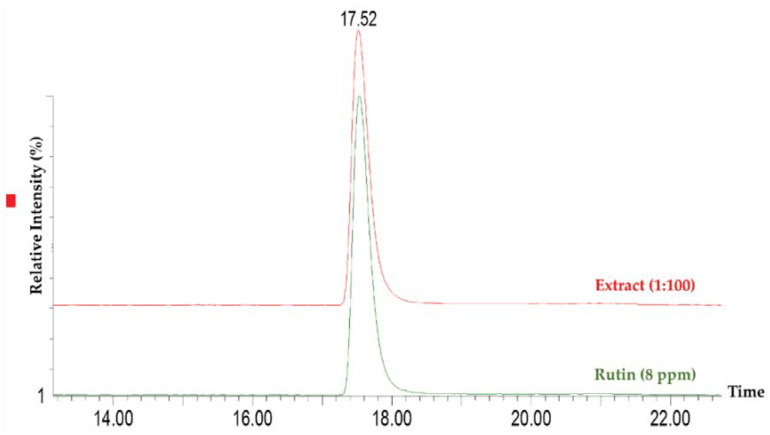
MRM overlay chromatograms of rutin in the extract with a standard solution.

The entrapment capacity (EC) was determined for extract-loaded ethosomes using rutin as reference (Figure 5). Rutin is the major phenolic compound of this extract (74.93 ± 17.00 mg/g of extract). This observed value is in accordance with the values found in literature [21,22,23]. In the current study, the ethosomes presented an EC of 73.91 ± 24.80% (*n* = 20) which might be a good indicator of the presence of stable interactions between extract and SPC. Besides other factors, the EC is generally dependent on the composition of ethosomes [24,25,26]. In previous works with similar composition of ethosomes, the value ranged between 65% and 71% of rutin [26,27,28], similar to our value. 

### 2.5. Characterization of Rutin and Ethosomes Complexes

**Figure 6:**
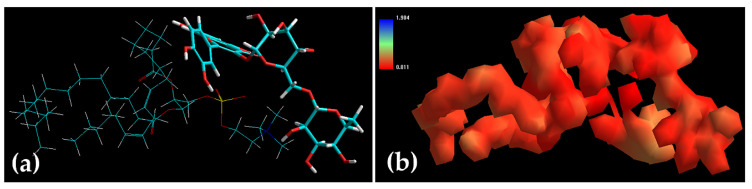
Representation of (**a**) geometrical preferences (**b**) electrostatic molecular graph after molecular mechanics simulations in vacuum (**a**,**b**) SPC-Rutin [Colour code for elements: C = cyan; H = white; O = red; N = blue]. Rutin: tube rendering; SPC: stick rendering.

Table 1 displays the inherent molecular energy attributes for various biomolecular complexes (SPC-rutin), while Figure 6 presents the corresponding geometrical positions. Here, the target was the major compound of the extract, rutin. In terms of the overall energy, the SPC-rutin complex was stabilized by the Van der Waals energy and it was accompanied by dihedral and electrostatic destabilizations. Interestingly, this comparatively destabilization of SPC-rutin molecular complex (∆E ≈ −6 kcal/mol) may provide enhanced diffusivity of rutin across the lipidic matrix, in comparison to the polymer-rutin complexes in a previous study performed by our group, where poly-glycolic-lactic acid-rutin and poly-caprolactone-rutin revealed higher molecular complex (∆E ≈ −20 kcal/mol and −18 kcal/mol, respectively) [4]. These chemical interactions might somehow justify the extract entrapment of around 70%, i.e., the interactions are not too strong in terms of energy.

### 2.6. In Vitro Release Studies of Extract from Ethosomes 

**Figure 7:**
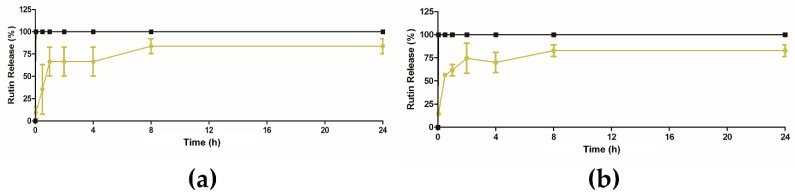
Percentage of rutin release free extract (black line) and extract-loaded ethosomes (yellow line), (**a**) in PBS pH 5.5, (**b**) in PBS pH 7.4.

The release profile of extract from ethosomes was performed using PBS at two different pHs, at pH 5.5 which is similar to the pH of the skin [2], and at pH 7.4 which corresponds to the biological pH of blood [29]. The assays were performed at controlled room temperature (RT ≈ 25 °C). The obtained results are displayed in Figure 7. The release studies showed that the extract-loaded ethosomes presented the same release profiles, independently of pH, achieving 83.8 ± 8.3% and 82.8 ± 6.4%, for PBS pH 5.5 and pH 7.4, respectively. This behavior was quite different from the free extract where its complete solubilization instantaneously occurred after 5 min. Here, the extract encapsulation into ethosomes led to a slight delay, which can favor the extract release in the target area. This retention time can be additionally modulated after inclusion in Carbopol gel as it was already reported in literature [30,31]. This effect is still controversial; some studies showed a slower release of the encapsulant but other described the same profile after inclusion in a semi-solid dosage form [32]. As a representative example, a very recent work described the preparation of ethosomes with *Achillea millefolium* L. extract. In the permeation study, ethosomes alone or after inclusion of Carbopol had the same behavior. Thus, further studies should be done with aim to verify this hypothesis.

### 2.7. In Vitro Collagenase Inhibition Activity of Extract after Encapsulation

**Figure 8:**
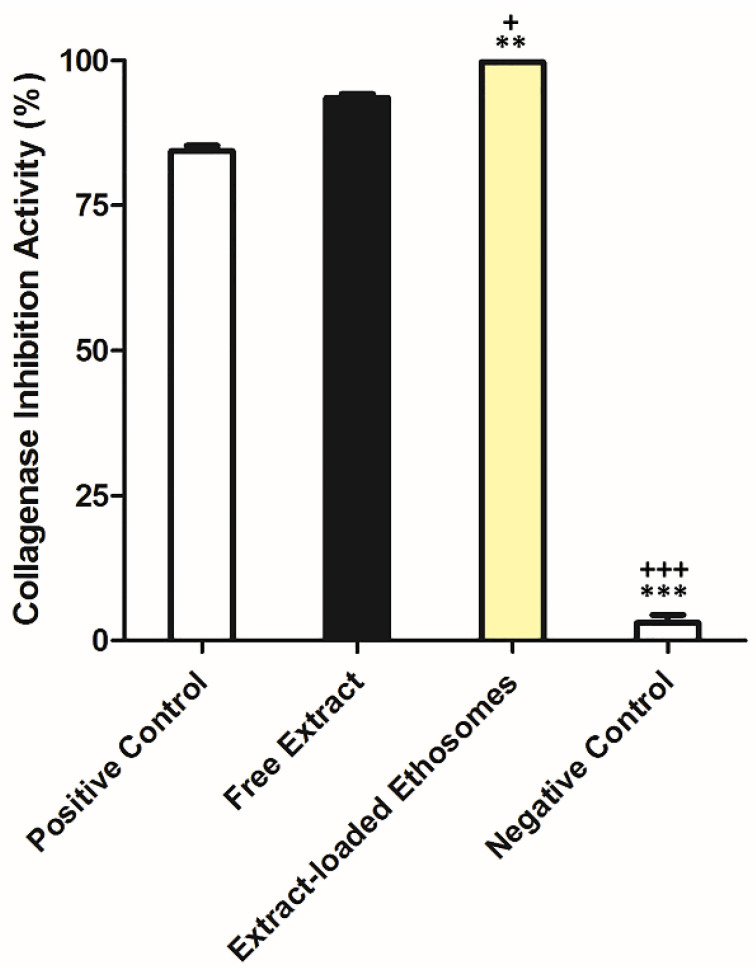
Coll inhibition activity of samples (1 mg of sample/mL, mean ± S.D., *n* = 3, ** *p* < 0.01 and *** *p* < 0.001, when compared to epigallocatechin gallate (EGCG, positive control—250 µM) and + *p* < 0.05 and +++ *p* < 0.001, when compared to free extract).

The results obtained for the in vitro collagenase inhibition activity are showed in Figure 8. The free extract (93.57 ± 0.61%) presented higher Coll inhibition activity the positive control (84.36 ± 0.91%). At equivalent concentration of the extract, extract-loaded ethosomes had the highest Coll inhibition activity (99.67 ± 0.09%). 

This enzyme is generally involved in some skin diseases or disorders, being associated with the degradation of the collagen-rich extracellular matrix (ECM). Furthermore, there is a relationship between Coll-assisted ECM breakdown and tumor invasion [5]. The inhibition of this enzyme can prevent skin disorders or contribute for the treatment of these diseases. Among the phytocompounds previously identified in this extract, this inhibition can be related to the presence of naringenin [33]. This value was higher than some values described in previous studies. As an example, Zofia et al. tested different extracts in terms of Coll inhibition. Inhibition ranged between 10–40% to *Meum athamanticum* L., less than 30% to *Centella asiatica* L. and varied between 20–75% to *Aegopodium podagraria* L. [34]. 

### 2.8. Preliminary In Vitro Safety Assessment of Extract in Ethosomes

**Figure 9:**
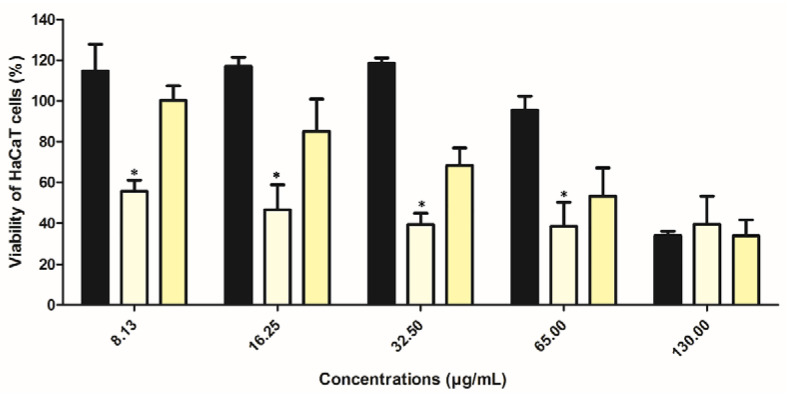
Cell viability (%) of HaCaT following 48 h incubation of free extract (black columns), empty (light yellow columns) and extract-loaded ethosomes (dark yellow columns). Tested concentration ranged from 8–130 µg of rutin/mL (%, mean ± S.D.; *n* = 6; * *p* < 0.05, when compared with free extract). Empty ethosomes were used at equivalent concentration of loaded ethosomes.

One important parameter in any formulation development is the safety [4]. Cell viability of HaCaT cells was performed for free extract as well as empty and extract-loaded ethosomes. The tested concentrations ranged from 8.13 to 130.00 µg of rutin/mL, and results are depicted in Figure 9. A dose-dependent effect for free extract and loaded ethosomes was observed but this tendency was not observed for empty ethosomes. This reduction of cell viability observed in the ethosomes (empty or loaded) is possibly due to the presence of ethanol, which was not present in the extract. Besides the presence of ethanol, the ethosomes sizes by itself can be another possible reason, since they can act as physical barriers and thus interfere with cell growth. This observation is already well documented in several previous studies. 

A wide number of published works have stated that ethosomes are safe to be applied in humans and animals, presenting an excellent skin tolerability [35]. In fact, SPC presents a very high tolerability and biocompatibility [36]. In terms of in vitro scenario, keratinocyte (HaCat) cells represent 95% of epidermal cells [4,37], being more sensible than skin by itself in a real scenario [4,38]. Thus, in principle, there should be no concerns in terms of its application in humans as further section confirmed it.

### 2.9. Preliminary Stability Test of Semi-Solid Formulation with Free Extract and Ethosomes

#### 2.9.1. Heating and Cooling Testing

The organoleptic characteristics, pH and viscosity of each sample were evaluated over one week for heating and cooling tests, with analyses in four different times (immediately after the incorporation of the ethosomes into the gels, on the second, fourth and last day). Samples of gel with extract (Gel + E), gel with empty ethosomes (Gel + Etho) and extract-loaded ethosomes (Gel + E-Etho) were compared to the gel (Carbopol^®^ 940 as control). This study reports for the first time that all these combinations were studied by diverse parameters and for different assays over time.

Concerning the Carbopol^®^ 940, no phase separation (PS) was observed over time, after inclusion of extract, empty ethosomes and loaded ethosomes. The organoleptic characteristics did not change over time, with maintenance of similar colour for each sample. The pH values were registered for each sample, and a stable pH was verified for all samples, with slight variations (1.04 maximum) over time. These small changes of pH did not have any impact on the consistency of the gel, which is in conformity with the results reported by Islam et al. [39]. Viscosity is another main parameter of a potential semi-solid formulation for topical use. This parameter was measured for all samples over the time and the values are registered in Table 2. A decrease of viscosity over time was noticed. The comparison between the gel (control) and the Gel + E revealed a statistical difference of *p* < 0.001 on day 0 and 4, but for the other time-points no difference was observed. The data suggested that the addition of extract led to an initial increase of viscosity, but after the second day, a decrease of the value of this parameter was registered, and this tendency was maintained over time. Concerning the samples of empty and extract-loaded ethosomes, it was verified a decrease of viscosity, which was maintained over time. This event can be a consequence of the addition of ethosomes in a solution. Regarding the free extract, it was included in gel in powder.

#### 2.9.2. Centrifugation Stress of Semi-Solid Formulation with Free Extract and Ethosomes

The results of the organoleptic characteristics showed a normal appearance before and after centrifugation for all the samples. The pH values after the centrifugation ranged between 5.39–7.00, for Carbopol gel. In general, the samples presented similar pH before and after the centrifugation. Concerning viscosity, it was observed a decrease after centrifugation as can be seen in Table 2. Results suggested that for all samples, the addition of extract and ethosomes (empty or loaded) decreased the viscosity of the gel after centrifugation.

### 2.10. Accelerated Stability of Semi-Solid Formulation with Free Extract and Ethosomes

#### 2.10.1. Tests Cycles of Heating and Cooling

Organoleptic characteristics observations revealed a normal appearance over time for all samples. The addition of extract into the gel decreases the pH of the gel and this behavior was maintained over the assay. On the other hand, the Gel + E-Etho led to an increase of pH value in the first moment and that was maintain until the end of assay. In terms of viscosity, samples of gel (control) and Gel + E presented a decrease at the end of the study. On the contrary, the sample of Gel + E-Etho presented a slight increase of viscosity over time and with the cycles of heating and cooling. Thus, concerning to viscosity this assay suggests that these samples present some vulnerability with the exposition to different temperature exposition.

#### 2.10.2. Stability Test over 14 Days

Organoleptic characteristics observations revealed a normal appearance over time for all samples under the different exposition temperatures. The addition of extract and extract-loaded ethosomes led to a slight decrease of the pH value that was kept over the assay, which suggest a stability of the samples under RC. Concerning the RT, the addition of extract or extract-loaded ethosomes increased the pH value and this difference was maintained over time. At AC, the addition of extract and extract-loaded ethosomes increased the pH value and this difference was also kept over time. The influence of temperature in the pH of the gel was evident, like previous study demonstrated [40]. The viscosity was measured for all samples in Carbopol^®^ 940 gel; it was observed an increase of viscosity over time at RC and RT for Gel + E-Etho but a decrease when exposed to AC and AC*, suggesting a direct correlation with the temperature to the Gel + E. Nevertheless, no correlation was noticed to the Gel (control) and to Gel + E-Etho. 

#### 2.10.3. Temperature Cycles

The temperature cycles have no interference with organoleptic parameters and pH values, but regarding the viscosity, it was verified a decrease of values at the end of assay, that was more evident for the Gel + E-Etho. With the increase of temperature, the molecular vibrations might have also increase, changing polymer entanglement [32]. The change can be also due to the syneresis of the sample by itself, presence of ethanol and other compounds from the loaded ethosomes. This behavior was already reported in previous studies described in the literature [41,42].

### 2.11. Stability over Three Months

Organoleptic characteristics observations were registered over three months, every month, under three different temperatures (RC, RT and AC). The results did not reveal any change. The results for pH showed a decrease of difference over time with the increase of temperature for some of the samples, probably due to the release of some phytocompounds of the extract entrapped into the ethosomes. Regarding the viscosity, this parameter changed in the presence of extract, ethosomes, the presence of both and with temperature. This variation was already observed in previous studies. As an example, Dave et al. developed several ethosomes with gel formulations, but the most similar formula with this study revealed a viscosity of 7063 ± 2.8 cP [43]. Another example revealed that the addition of ethosomes in Carbopol gel affects its flow and viscoelastic behavior [44]. These authors suggested that the increase of temperature led to a decrease of viscosity, a consequence of the loosing of polymer entanglement at higher temperatures. 

### 2.12. Rheology of Semi-Solid Formulation with Free Extract and Ethosomes

**Figure 10:**
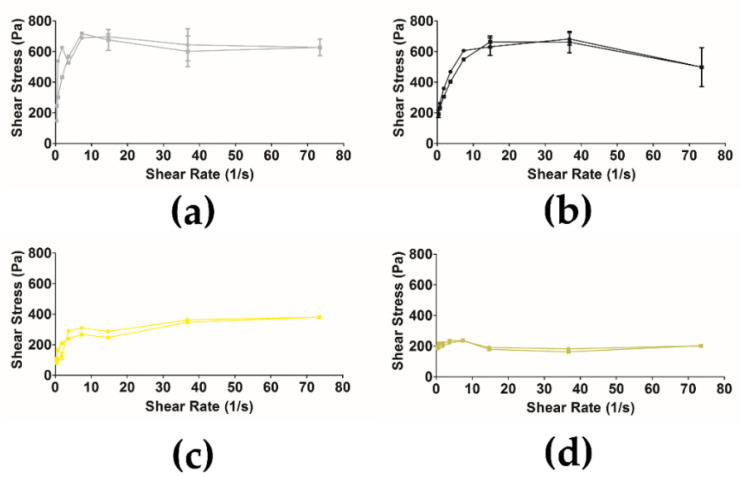
Rheology profile of samples (•) when increase the speed and (▪) when decrease the speed (**a**) Gel (control), (**b**) Gel + E, (**c**) Gel + Etho, and (**d**) Gel + E-Etho (mean ± S.D.; *n* = 3).

The rheology is important to characterize the stability of samples as well as to develop consumer acceptable final products [45]. This parameter allows to understand the rheological nature of the formulation, to perform studies of quality control of the effect of parameters like formulation, storage time and temperature on the quality of the formulation and to assess a product with regard to actual usage, such as spreading and adherence to the skin and removal from a tube or a jar. Profiles of all samples are presented in Figure 10. The results suggest that these samples present a shear thinning pseudoplastic behavior, which is according to the literature [46]. All tested samples consisted of hydrogels that are classified as non-Newtonian systems, exhibiting a non-linear relationship between stress and shear rate. 

### 2.13. Texture of Semi-Solid Formulation with Free Extract and Ethosomes

**Figure 11:**
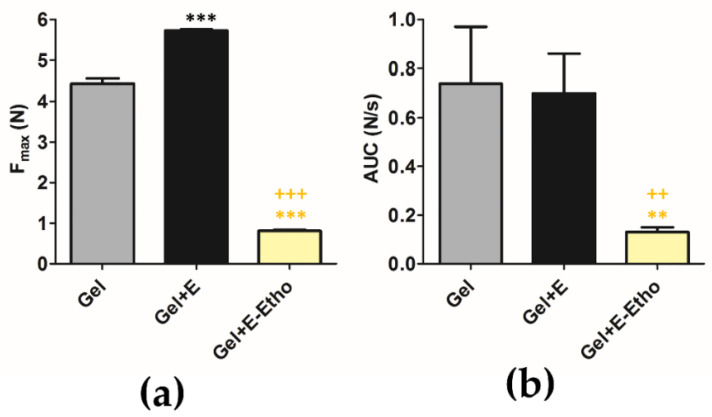
The (**a**) peak force of displacement (F_max_, N) and the (**b**) area of the peak (AUC, N/s) obtained from the force versus time curves. The results are presented regarding the mean ± S.D.; *n* = 3 (** *p* < 0.01 and *** *p* < 0.001 when compared with gel (control); ^++^ *p* < 0.01 and ^+++^ *p* < 0.001, when compared with Gel + E).

It is generally accepted that the ideal characteristics of a fine semi-solid formulation for a better consumer acceptance are a good skin spread ability, an easy removal of product from the package and a good skin adhesion [47]. So, the aim of this study was to evaluate the textural properties of the different formulations concerning the physical gel structure [48]. The samples were analyzed in triplicate and the maximum peak force of displacement, also named hardness (F_max_, N) and area of the peak (AUC, N/s) are displayed in Figure 11. It was observed that the Gel + E seems to be more adhesive than the Gel + E-Etho, probably due to the presence of ethanol and dilution after ethosomes inclusion. 

### 2.14. Skin Compatibility Test of Semi-Solid Formulation with Free Extract and Ethosomes

The obtained results are presented in Table 3. Both samples showed very good skin compatibility. No reaction was detected for both samples in all the volunteers, confirming the safety of this formulation. The decrease of adhesion of the Gel + E-Etho observed in the texture analysis suggests that this change did not have any impact after topically delivered. Other properties are also important to achieve a suitable and effective topical delivery, such as the thixotropic properties and spreadability of the gel.

## 3. Materials and Methods

### 3.1. Materials

#### 3.1.1. Plant Material

*S. nigra* L. flowers (elderflowers) were supplied by *Régiefrutas—Cooperativa Agrícola de Interesse Público Távora-Varosa*, CIPRL, collected from commercial crops at Tarouca, Beira Alta, Portugal (lat. 40° 59′ 06″ N; long. 7° 37′ 03″ W; 695 m alt.) in May 2019.

#### 3.1.2. Chemicals

Phosphatidylcholine (l-α-Phosphatidylcholine, also named soybean hosphatidylcholine, SPC) was supplied by Sigma Aldrich (St. Louis, MO, USA). Oleic Acid was acquired by Fluka Chemika (Buchs, Switzerland). Coll from Clostridium histolyticum type IA, Dulbecco’s Modified Eagle’s Medium—high glucose (DMEM), quercetin, thiazolyl blue tetrazolium bromide (MTT), Span 20^®^, Nile Blue A and ascorbic acid were supplied by Sigma Aldrich (Steinheim, Germany). Fetal bovine serum (FBS) purchased from Biowest (Riverside, CA, USA). L-Glutamine was supplied by Lonza (Leuven, Belgium). Rutin was acquired from Extrasynthese (Genay, France). Methanol was purchased from António M. S. Cruz (Amadora, Portugal) and sodium chloride (NaCl) from José M. Vaz Pereira (Benavente, Portugal). Epigallocatechingallate (EGCG), *N*-[3-furyl-acryloyl]-Leu-Gly-Pro-Ala (FALGPA) were purchased from Panreac (Barcelona, Spain). Tricine buffer was acquired by VWR (Leuven, Belgium). Carbopol 940^®^ was purchased from Fagron (Barcelona, Spain). Folin reagent was supplied by Merck (Darmstadt, Germany). High performance liquid chromatography (HPLC) grade acetonitrile and formic acid were obtained from Chem-Lab NV (Zedelgem, Belgium). Milli-Q water (18.2 MΩ cm^−1^ resistivity) was obtained from a Millipore-Direct Q3 UV system (Millipore^®^, Burlington, MA, USA). All other chemicals were of analytical grade.

#### 3.1.3. Cell Lines

The HaCaT cell line was supplied by Cell-Line-Service (cat: 300493, Eppelheim, Germany). 

### 3.2. Methods

#### 3.2.1. Extraction

The extract was obtained from fresh elderflowers, using methanol as solvent through ultrasonication method (Sonorex Super RK 510 H; Bandelin, Berlin, Germany) for 1 h. The procedure was repeated three times until complete extraction [4,5,49]. The extract was then filtered, and the methanol was removed by rotary evaporation (VV2000 rotary evaporator from Heidolph, Apeldoorn, The Netherlands).

#### 3.2.2. Ethosomes Preparation Method

To produce ethosomes, 2% of SPC (*w*/*v*), 40% of ethanol (*v*/*v*) and 58% of MilliQ water (*v*/*v*) were used. Ethosomes then were centrifuged at 10,460× *g* (Centrifuge, Sigma Laborzentrifugen, Osterode am Harz, Germany) for 10 min at room temperature (RT) and then resuspended in MilliQ water. For the extract-loaded ethosomes, the same method was performed, and the amount of extract used was according to the proportion 1:1, *w*/*w*, lipid:extract. Empty and extract-loaded ethosomes were stored at 4 ± 2 °C—refrigeration conditions (RC). The ethosomes stored at these conditions were further used in all the assays.

#### 3.2.3. Lipid Quantification 

The lipid content of vesicles was determined using the modified colorimetric method described by Rouser et al. [50,51]. Briefly, the samples (in triplicate) with an amount of phosphate between 20 and 80 nmol (sample volume below 100 μL) were cooled and after that 0.3 mL of perchloric acid (70–72%) was added. Furthermore, all tubes were heated at 180 °C for 45 min, in order to convert all the organic lipid phosphate to its inorganic form and the samples were cooled to RT. Subsequently, 1.0 mL of H_2_O and 0.4 mL of hexa-ammonium heptamolybdate solution [1.25% (*w*/*v*)] followed by 0.4 mL of ascorbic acid solution [5% (*w*/*v*)] were added. It was obtained a blue color solution due to the reduction of ascorbic acid over the heating process. In the end of method, the absorbance of all samples was recorded at 797 nm) in a UV-mini 1240 spectrophotometer (Shimadzu, Kyoto, Japan) [51]. In parallel, a calibration curve was also prepared with amounts of phosphate ranging from 20 and 80 µmol/tube, also in triplicate. All tubes were heated (180 °C) in a heating block until dryness.

#### 3.2.4. Physical Characterization of Ethosomes: Size, Surface Charge and pH 

Mean size, PdI, zeta potential and pH of empty and extract-loaded ethosomes were evaluated for up to 12 months [52]. Zeta potential of the ethosomes was measured in diluted NaCl (0.1 M) solution (1:10, *v*/*v*). The size, PdI and zeta potential were measured in diluted samples (1:16, *v*/*v*) by DLS, using a Malvern Zetasizer Nano-S and Nano-Z (Malvern Instruments, Worcestershire, UK). Results were expressed as mean of measurements in triplicate (*n* = 3). Stability study at different temperature was performed using three different temperatures (4 ± 2 °C—RC, 25 ± 5 °C—RT and 40 ± 2 °C—AC). Results were expressed as the mean ± S.D. (*n* = 3). The pH of the obtained suspension of ethosomes was measured using a pH electrode meter (827 pH Lab, Metrohm, Herisau, Switzerland), calibrated every day of measurements, with buffer solutions pH 4.00 ± 0.02 and 7.00 ± 0.02 (20 °C) ST (Panreac).

#### 3.2.5. Histochemical Characterization of the Lipidic Constitution of Ethosomes and Study of their Morphology

The lipidic constitution of ethosomes was histochemically demonstrated by Nile blue A, a stain used for detection of acidic and neutral lipids [53]. Microscope slides were prepared with a drop of the vesicular suspension, previously stained with an aqueous solution of 0.1% Nile blue A. Observations were carried out in bright field and in fluorescence with an BX51 light microscope (Olympus, Tokyo, Japan) equipped with a Nomarski and an epifluorescence condenser. For fluorescence, a filter set for green light with excitation at 535/30 nm and emission at 580 nm was used. Images were recorded digitally.

The morphological characteristics of the particles were investigated by scanning, transmission and atomic force microscopy techniques (SEM, TEM and AFM, respectively). For SEM, aliquots (20 µL) of the vesicular suspensions were carefully dispersed on round glass coverslips coated with poly L-lysine and previously attached to the microscope stubs. The samples, after dried in a desiccator, were sputter-coating with gold and observed with a 5200LV scanning electron microscope (JEOL Ltd., Tokyo, Japan) at an accelerating voltage of 20 kV. Images were recorded digitally. For TEM, aliquots (10 µL) of NPs suspensions were placed on Formvar/carbon coated grids and the excess solution was removed with a filter paper. Then, the material was negatively stained with 1% of uranyl acetate and left in room conditions for air-drying. Observations were carried out on a 1200EX transmission electron microscope (JEOL Ltd., Tokyo, Japan) operating at 80 kV and images were recorded digitally.

For the atomic force microscopy (AFM) analysis, the samples were centrifuged at 7378× *g* (Sigma Laborzentrifugen, Osterode am Harz, Germany) for 10 and 20 min, for empty and extract-loaded vesicles, respectively, followed by resuspension in water (in half of the previous volume) [54]. AFM uses a diluted sample, 1:2 without any pre-treatment. A freshly cleaved mica surface was used to place a drop (≈40 μL of sample), being allowed to adsorb for around 30 min [54]. After drying using a stream of N2, samples were analyzed in intermittent mode (Multimode 8 HR Microscope, produced by Bruker, Billerica, MA, USA) [54]. Images were acquired in ambient conditions (≈21 °C), through the use of etched silicon tips with a resonance frequency of around 320 kHz (NCHV, Bruker), at a scan rate near to 1.3 Hz [54]. All images were recorded digitally.

#### 3.2.6. Entrapment Capacity of Extract in Ethosomes

To determine the EC of the elderflower extract, the samples were analyzed by HPLC-MS/MS aiming at quantifying rutin, the major compound of the methanolic extract. The Waters^®^ Alliance 2695 HPLC system (Waters^®^, Dublin, Ireland) equipped with an autosampler, quaternary pumps, column furnace, and a diode-array detector (DAD) Waters 996 DAD (Waters^®^) was used to performer the assays. Chromatographic analyses were carried out using a LiChrospher^®^ 100 RP-18, 5 μm (250 × 4 mm) column at 35 °C. A mixture of formic acid (0.5% *v*/*v* in ultrapure water) (eluent A) and 0.5% formic acid in acetonitrile (eluent B) at a flow rate of 0.3 mL/min, were used as a mobile phase. The initial conditions were maintained for 20 min as a re-equilibration step. The gradient was: 5% B (0–10 min), 15% B (10–30 min), 20% B (30–45 min), 20% B (45–65 min), 54% B (65–95 min), 63% B (65–110 min), and 5% B (110–115 min). The total running time was 135 min and the injection volume 20 µL. The HPLC system was coupled to a triple quadrupole mass spectrometer MicroMass Quattromicro^®^ API (Waters^®^, Dublin, Ireland) equipped with an electrospray ionization source (ESI). 

MS/MS conditions were optimized for the identification and quantification of rutin. The electrospray ion source (ESI) was set to operate at 120 °C in negative mode, using a capillary voltage of 2.5 kV, cone voltage of 45 V and collision energies of 32, and 34 eV. High purity nitrogen was used as drying and as a nebulizing gas. The collision gas used was the ultra-high purity argon. Analyses were performed in multiple reaction monitoring mode (MRM), selecting the one product ion with the highest signal as the monitored transitions for quantification (MRM1, 609.00 > 300.00) and confirmation (MRM2, 609.00 > 271.00) purposes. MassLynx Version 4.1 software (Waters) was used for instrument control, data acquisition, and data processing.

The *EC* was determined by analyzing the rutin present in the supernatant—indirect method). *EC* was determined by the Equation (1):

(1) $$EC=\left(\frac{Total\;amount_{major\;bioactive\;compound}-Amount\;free_{major\;bioactive\;compound}}{Total\;\;amount_{major\;bioactive\;compound}}\right)\times 100$$

#### 3.2.7. Rutin and Ethosomes Complex Simulation

The reactional profile of extract and ethosomes system was assessed in silico via molecular mechanics simulations and the ensuing energetic-geometric stabilization provided an insight into the antioxidant potential of ethosomes. To analyze the mechanism governing the Coll potential of the ethosomes, energetic and geometrical stabilization of the drug-lipid molecular complexes were conducted using atomistic simulations (HyperChemLite Molecular Modelling Software, Hypercube Inc., Gainesville, FL, USA). The structures of SPC and rutin were generated as natural bond angles. The individual molecules and the biomolecular complexes (SPC-rutin) were energy minimized and optimized employing MM3 Force Field algorithm which was further accompanied by a Polak–Ribiere Conjugate Gradient method until an RMS gradient of 0.001 kcal/mol was achieved [55,56].

#### 3.2.8. In Vitro Release Studies

Extract-loaded ethosomes were previously lyophilized over 24 h at −50 °C (freeze–dryer model, Edwards, Edwards, CO, USA). Afterwards, they were solubilized into phosphate buffer solution (PBS) (USP41) pH 5.5 (10 mL) to simulate human skin pH and stirred (200 rpm) at 32 °C in a multiplate stirring plate. Sink conditions were assured over the whole assay. Aliquots of the release medium were collected at appropriate time intervals (5 and 30 min, 1, 2, 4, 8 and 24 h), and replaced immediately with fresh buffer. The same assay was performed in PBS at pH 7.4 (USP41). As reference, rutin was previously identified as the major flavonoid of the extract [5], therefore a standard calibration curve was performed with rutin solution in PBS buffer pH 5.5 or 7.4, depending on the assay. Extract concentration at each time point was determined using HPLC-DAD- MS/MS. 

HPLC-DAD-MS/MS assays were carried out on a Waters^®^ Alliance 2695 HPLC system (Waters^®^) coupled to a 2996 Photodiode Array Detector and a Micromass^®^ Quattro Micro triple quadrupole (TQ) (Waters^®^). These analyses were performed at 35 °C on a LiChrospher^®^ 100 RP-18 (250 × 4 mm, 5 μm) column. The mobile phase used was the same as described in Section 3.2.6. The elution program consisted of 20% B (0–5 min), with increase to 90% B in 10 min, 90% B (15–20 min), and with a decrease to 20% B in 1 min, and ultimately the initial conditions were maintained for 20 min as a re-equilibration step. Total run time was 40 min and the injection volume 10 µL. MS/MS conditions were already described in Section 3.2.6.

#### 3.2.9. In Vitro Collagenase Inhibition Activity

The protocol used was already described in previous works [4,5,57]. This assay was performed in 50 mM tricine buffer (pH 7.5) enriched with 400 mM NaCl, and 10 mM CaCl_2_. The FALGPA (substrate) was dissolved in tricine buffer (2 mM) whereas Coll from *Clostridium histolyticum* (EC.3.4.23.3) was dissolved in a buffer at an initial concentration of 0.8 Units/mL and according to the supplier’s activity data. Negative controls were the respective sample solvent. The absorbance was measured at 405 nm after the substrate addition over 10 min using a microplate reader (Thermo Scientific Multiskan FC, Shanghai, China). The positive control (EGCG) was used at a concentration of 250 μM. This compound has been reported as being a strong inhibitor of collagen degradation [58]. The Coll inhibition (%) was determined using the Equations (2) and (3):

(2) $$Velocity\;reaction\;of\;control\;or\;inhibitor=\frac{Corrected\;Abs}{time\;\left(min\right)}$$

(3) $$Collagenase\;inhibition\;activity\;\left(\%\right)=100-\left(\frac{100\times Velocity\;reaction\;of\;inhibitor}{Velocity\;reaction\;of\;control}\right)$$

For the Coll activity, the absorbance decrease was calculated using the Equation (2) for the velocity reaction of negative control (ΔAbs_405nm_/min) and after to determine the Coll inhibitions activity, it was used the Equation (3) [4,5,57].

#### 3.2.10. In Vitro Safety Assessment 

The in vitro safety of the free extract, empty and extract-loaded ethosomes were evaluated using the MTT assay in the human keratinocyte cell line (HaCaT, cell line). These cells were seeded in 96-wells plate at a density of 5 × 10^4^ cells/mL in DMEM with high-glucose (4500 mg/L), supplemented with 10% FBS, and 100 IU/mL of penicillin and 100 µg/mL streptomycin [(Pen/Strep, 1%, *v*/*v*)], and allowed to grow for 24 h in a humidified chamber at 37 °C in a 5% CO_2_ atmosphere [59]. For this assay, the medium was removed and samples at concentrations ranging from 8.13–130.00 µg of rutin/mL (free extract and equivalent concentrations of extract into ethosomes, according to the results obtained for EC were prepared and added to HaCaT cells. After 48 h, the samples were removed, and the cell monolayers were washed with PBS. Then, 50 μL of MTT at 0.5 mg/mL was added to the cells and the plates were incubated for 4 h in a humidified chamber at 37 °C and 5% CO_2_ atmosphere. After this incubation time, 100 μL of DMSO was added to each well to solubilize the formazan crystals. The absorbance (Abs) was measured, and cell viability was calculated using the same Equation (4):

(4) $$Cell\;Viability\;\left(\%\right)=\frac{Abs_{t}}{Abs_{c}}\times 100$$

where *Abs_t_* is the absorbance of the sample and *Abs_c_* the absorbance of the control.

#### 3.2.11. Inclusion of Extract-Loaded Ethosomes in a Semi-Solid Formulation

Carbopol^®^ 940 gel was prepared based on previous literature with slight modifications [60]. Briefly, 500 mg of Carbopol^®^ 940 was dispersed in water under stirring (400 rpm) until complete solubilization. Then, 0.2% of methyl 4-hydroxybenzoate and 0.02% of propyl-4-hydroxybenzoate were added under constant stirring. Finally, NaOH was added to Carbopol (0.4 g of NaOH per gram of Carbopol) under magnetic stirring. This last reagent was responsible for the gelling effect.

**Figure 12:**
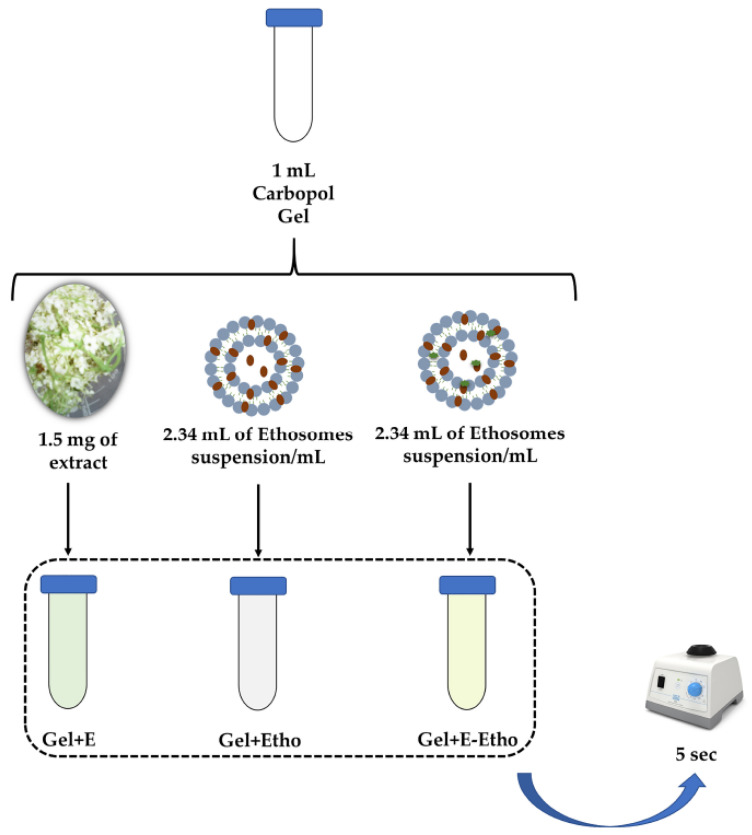
Schematic representation of inclusion of Extract-Loaded Ethosomes in a Semi-Solid Formulation.

The resultant Carbopol^®^ 940 gel was then characterized. A total of four different samples were tested: gel only (control); gel + Extract (Gel + E); gel + empty ethosomes (Gel + Etho) and gel + extract-loaded ethosomes (Gel + E-Etho), as schematic illustrated on the Figure 12. Incorporation of extract was performed according HPLC values (Section 3.2.6). Specifically, 1.5 mg of extract was mixed with 1 mL of Carbopol gel, followed by vortexing 5 s. Incorporation of ethosomes was performed by adding 2.34 mL of ethosomes suspension/mL of Gel to a tube, followed by same vortexing. 

#### 3.2.12. Characterization of the Semi-Solid Formulation for Skin Delivery of *S. nigra* Extract

- Organoleptic Characteristics

The obtained gels with and without ethosomes were characterized in terms of aspect (colour and homogeneity) and odor. The organoleptic characteristics were evaluated by direct observation of samples. When the physical appearance was maintained, the samples were classified as normal whereas they were classified as PS when a phase separation was observed.

- pH Measurement

Measurement of the pH was performed at RT in triplicate using potentiometer (827 pH Lab, Metrohm, Herisau, Switzerland). This equipment was daily calibrated with buffer solutions pH 4.00 ± 0.02 and 7.00 ± 0.02 (20 °C) (Panreac).

- Viscosity Measurement

The viscosity was measured using a DV-I + Viscometer (Brookfield Engineering Labs. Inc., Middeborough, MA, USA) with the n° 4 needle and using a speed rate of 3 rpm.

- Preliminary Stability Assays of Semi-Solid Formulation with Free Extract and Ethosomes

The following tests were performed in all samples, i.e., with and without empty and extract-loaded ethosomes as previously reported by Reis et al. [61] and according to the International Guideline ICHQ1A (R2) [62]. The preliminary stability assays included the heating and cooling, as well as the centrifugation stress:

(1) Heating and Cooling

Samples were submitted to heat-freeze cycles [25 ± 2 °C in an oven (24 h), then cooled to −5 ± 2 °C in a freezer (24 h)], over one week. Frozen samples were allowed to melt and cool down to RT prior measurements. Samples were analyzed at 48, 96 and 168 h in terms of organoleptic characteristics, pH measurement and viscosity, as previously presented [61].

(2) Centrifugation Stress

This assay was adapted from Reis et al. (2015) [61]. Each sample (approximately 6 g) was exposed to a 50 °C water bath (Heidolph MR3001, Heidolph Instruments, Schwabach, Germany) and, subsequently, centrifuged at 1077× *g* for 30 min (Beckman Gpr Refrigerated Centrifuge Rotor, Indianapolis, IN, USA). The parameters of organoleptic characteristics, pH measurement and viscosity, were verified before and after centrifugation.

- Accelerated Stability Assays of Semi-Solid Formulation with Free Extract and Ethosomes

The following tests were performed in empty and extract-loaded ethosomes as previously reported [61] and under Guideline ICHQ1A (R2) [62].

(1) Tests Cycles of Heating and Cooling

Samples were incubated at 45 ± 2 °C in the oven and cooled in the freezer at −10 ± 2 °C (cycles of 24 h at each condition) for twelve days. Samples were analyzed every two days. This analysis included the evaluation of the parameters referred above (organoleptic characteristics, pH measurement and viscosity).

(2) Stability Test over 14 Days

Samples were exposed to three different settings: refrigerated conditions (RC, −5 ± 2 °C); RT (20 ± 5 °C), and accelerated conditions (AC, 50 ± 2 °C and AC*, 40 ± 2 °C) for 14 days. In this case, two different ovens were used to evaluate different conditions, one of them in regular or normal oven at 50 ± 2 °C, and the other one in a climatic chamber at 40 ± 2 °C. After 3, 7 and 14 days, formulations were analyzed and the parameters, already mentioned evaluated.

(3) Temperature Cycles

Samples were exposed to water bath at 40 °C, with a controlled heating rate of 10 °C/30 min of up to 80 °C. After returning to RT, the samples were analyzed in the same parameters than the previous tests.

- Stability over Three Months

Samples were exposed to three different settings: RC (−5 ± 2 °C); RT (20 ± 5 °C), and AC (50 ± 2 °C) for 3 months. After 1, 2 and 3 months, formulations were analyzed and organoleptic characteristics, pH measurement and viscosity determined.

- Rheological Properties

The procedure was based on the method of Braden [63], with slight modifications. The same needle was used and the values of viscosity for different speeds starting with the lowest (0.3 rpm) and gradually increasing the speed until 60 rpm.

- Texture Analysis

The texture of all samples was assessed by the TA.XT.plus (Texture Analyzer Stable Micro Systems, Surrey, UK). This assay was done using the following characteristics: pre-test speed (0.50 mm/s); test speed (0.50 mm/s); post-test speed (10.00 mm/s); applied force (500.0 g); return distance (10.000 mm); contact time (10.00 s); trigger type (automatic) and trigger force (5.0 g). The estimated mean areas under the force-time curve were calculated for each sample, in triplicate analysis.

#### 3.2.13. Human Skin’s Compatibility Testing

The skin’s compatibility was performed by occlusive patch Finn Chamber^®^ standard [64] with some modifications made by Mazulli et al. [65]. A group of 12 subjects (n = 6 each group) with age between 18 to 70 years, female and male, with phototype (Fitzpatrick) I to IV and to all type of skin was randomized in two different groups: one group dosed with gel with extract and the other group dosed with gel with extract-loaded ethosomes. The goal of the present case was reached after a single application to the human skin. The tested samples were: the final formulation of Gel + E and Gel + E-Etho (20 µL each). The formulations were in contact with skin, under patch in the back, for 48 ± 5 h. The examination was carried out, visually under standard “daylight”, before patching on first day and about 15 min (or more if some redness appeared after patch removal), then 24 and 48 h after patch removal. All the tests were made according to the Declaration of Helsinki and received the approval of the local Ethics Committee. 

The mean daily irritation score (Mdis) was determined using Equation (5), where Idis represents the individual daily irritation score, which is obtained by the sum of the marks obtained for all the signs observed [66].

(5) $$Mdis=\frac{\sum Idis}{number\;of\;valid\;cases}$$

#### 3.2.14. Statistical Analysis

Results were expressed as mean ± standard deviation (S.D.). The results concerning the biological assays were expressed as mean ± standard error of the mean (S.E.M.). One-Way ANOVA for multiple comparisons was used to assess the significance of differences by Graph Prisma Version 5.03 (GraphPad Software, San Diego, CA, USA). Only it was considered the significant differences when *p* < 0.05. Two-Way ANOVA for multiple comparisons between all samples. Concerning to the results of incorporation of the extract-loaded ethosomes were expressed as mean ± S.D. Statistical analysis was performed using Two-Way ANOVA for multiple comparisons between control and different formulations by Bonferroni test, using GraphPad Prism 5.03. Results were considered significantly different when *p* < 0.05.

## 4. Conclusions

Ethosomes demonstrated to be a good nanocarrier for the *Sambucus nigra* L. flower extract, regarding the high EC. In addition, these nanocarriers showed a high value of Coll inhibition. After the incorporation in Carbopol gel, these results suggested to be a stable gel over the time, in terms of organoleptic characteristics, pH and viscosity at different temperatures of storage. This formulation was also safe for humans and thus it can be considered a promising topical formulation, attracting a wide interest from cosmetic industry.

## Figures and Tables

**Table 1:** Inherent energy attributes of SPC-rutin complexes calculated using static-lattice atomistic simulations (SLAS) in vacuum.

Energy	Rutin	SPC	SPC-Rutin	ΔE ^1^
Total ^2^	23.09	1.10	18.61	−5.58 ^8^
Bond ^3^	1.16	1.39	2.49	−0.05 ^8^
Angle ^4^	7.57	35.69	42.85	−0.41 ^8^
Dihed ^5^	14.32	10.28	27.60	3.00 ^9^
vdW ^6^	0.04	−4.25	−14.35	−10.15 ^8^
Elec ^7^	0.00	−42.01	−39.97	2.04 ^9^

^1^ ΔE(A/B) = E(A/B) − [E(A) + E(B)]; ^2^ total steric energy for an optimized structure; ^3^ bond stretching contributions; ^4^ bond angle contributions; ^5^ torsional contribution arising from deviations from optimum dihedral angles; ^6^ Van der Waals interactions; ^7^ electrostatic interactions; ^8^ values represent the structure stabilizing contribution; and ^9^ values represent the structure destabilizing contribution.

**Table 2:** Stability data of the tested samples (Gel; Gel + E; Gel + Etho and Gel + E-Etho) at different testing conditions (mean ± SD, *n* = 3). OC means organoleptic characteristics, E means extract, Etho means ethosomes and E-Etho means extract-loaded ethosomes (N—normal, AC* at 40 ± 2 °C).

**Time (Days)**	**Gel**			**Gel + E**			**Gel + Etho**			**Gel + E-Etho**		
	**OC**	**pH**	**Viscosity**	**OC**	**pH**	**Viscosity**	**OC**	**pH**	**Viscosity**	**OC**	**pH**	**Viscosity**
**Preliminary Stability Testing (Heating and Cooling)**												
**0**	N	7.68 ± 0.01	134,050 ± 14,453	N	6.39 ± 0.03	159,800 ± 200	N	7.39 ± 0.02	183,933 ± 14,609	N	8.77 ± 0.01	155,533 ± 1922
**2**	N	7.65 ± 0.01	128,333 ± 1246	N	6.35 ± 0.21	123,933 ± 306	N	7.37 ± 0.02	129,667 ± 874	N	8.55 ± 0.04	28,200 ± 200
**4**	N	7.59 ± 0.02	124,867 ± 566	N	6.33 ± 0.12	107,333 ± 416	N	7.21 ± 0.03	100,067 ± 2055	N	8.50 ± 0.01	53,400 ± 872
**7**	N	7.45 ± 0.01	111,717 ± 58	N	6.13 ± 0.03	108,333 ± 643	N	7.27 ± 0.01	122,700 ± 200	N	8.45 ± 0.02	29,800 ± 200
**Preliminary Stability Testing (Centrifugation Stress)**												
**Before**	N	6.89 ± 0.01	192622 ± 518	N	6.60 ± 0.03	162,533 ± 306	N	5.38 ± 0.02	28,000 ± 200	N	7.11 ± 0.04	53,600 ± 529
**After**	N	6.96 ± 0.02	190933 ± 115	N	6.63 ± 0.01	150,733 ± 306	N	5.39 ± 0.03	27,867 ± 115	N	7.00 ± 0.01	49,000 ± 200
**Accelerated Stability Testing (Heating and Cooling)**												
**0**	N	7.58 ± 0.18	158,267 ± 2203	N	6.19 ± 0.02	156,933 ± 3252				N	7.50 ± 0.02	20,800 ± 529
**2**	N	6.72 ± 0.02	175,467 ± 25015	N	6.16 ± 0.08	133,800 ± 200				N	7.63 ± 0.04	38,733 ± 306
**4**	N	6.78 ± 0.02	193,800 ± 200	N	6.10 ± 0.02	98,600 ± 200				N	7.59 ± 0.02	31,200 ± 200
**6**	N	6.72 ± 0.02	133,400 ± 200	N	6.11 ± 0.01	128,933 ± 1890				N	7.61 ± 0.02	30,200 ± 200
**8**	N	6.73 ± 0.01	194,867 ± 5601	N	6.07 ± 0.04	62,600 ± 721				N	7.64 ± 0.01	40,867 ± 306
**10**	N	6.70 ± 0.01	114,600 ± 529	N	6.09 ± 0.17	57,800 ± 200				N	7.67 ± 0.04	25,000 ± 200
**12**	N	6.66 ± 0.02	96,467 ± 416	N	6.43 ± 0.16	73,467 ± 306				N	7.62 ± 0.02	28,533 ± 115
**Time (days)**	**Gel**			**Gel + E**			**Gel + Etho**			**Gel + E-Etho**		
	**OC**	**pH**	**Viscosity**	**OC**	**pH**	**Viscosity**	**OC**	**pH**	**Viscosity**	**OC**	**pH**	**Viscosity**
**Accelerated Stability Testing (14 Days)—RC**												
**0**	N	8.88 ± 0.14	168,333 ± 306	N	6.34 ± 0.01	104,000 ± 200				N	6.77 ± 0.03	33,467 ± 306
**3**	N	9.17 ± 0.08	66,533 ± 306	N	6.33 ± 0.02	151,200 ± 200				N	6.72 ± 0.01	42,200 ± 200
**7**	N	9.01 ± 0.06	68,000 ± 200	N	6.18 ± 0.01	118,333 ± 306				N	6.73 ± 0.03	41,333 ± 987
**14**	N	8.89 ± 0.02	50,200 ± 200	N	6.26 ± 0.17	99,933 ± 306				N	6.70 ± 0.02	64,733 ± 503
**Accelerated Stability Testing (14 Days)—RT**												
**0**	N	6.31 ± 0.06	194,533 ± 503	N	6.78 ± 0.02	167,200 ± 400				N	8.50 ± 0.01	21,800 ± 200
**3**	N	6.61 ± 0.02	185,467 ± 306	N	6.68 ± 0.03	138,200 ± 200				N	8.44 ± 0.02	29,400 ± 200
**7**	N	6.50 ± 0.03	121,200 ± 200	N	6.60 ± 0.04	109,600 ± 800				N	8.34 ± 0.01	129,400 ± 3470
**14**	N	6.54 ± 0.03	138,867 ± 416	N	6.55 ± 0.02	101,133 ± 306				N	8.03 ± 0.05	81,600 ± 4386
**Accelerated Stability Testing (14 Days)—AC**												
**0**	N	5.89 ± 0.04	159,267 ± 2444	N	7.42 ± 0.02	145,200 ± 200				N	8.02 ± 0.02	127,333 ± 503
**3**	N	6.15 ± 0.03	181,200 ± 200	N	7.35 ± 0.02	118,200 ± 200				N	7.79 ± 0.01	27,600 ± 200
**7**	N	6.21 ± 0.01	136,933 ± 306	N	7.08 ± 0.01	103,533 ± 611				N	7.78 ± 0.01	33,200 ± 200
**14**	N	6.27 ± 0.02	121,800 ± 200	N	7.01 ± 0.02	105,200 ± 200				N	7.67 ± 0.01	35,933 ± 306
Accelerated Stability Testing (14 Days)—AC *												
**0**	N	6.57 ± 0.01	158,667 ± 1617	N	6.74 ± 0.01	163,000 ± 1929				N	7.40 ± 0.01	71,533 ± 2759
**3**	N	6.60 ± 0.02	190,800 ± 721	N	6.77 ± 0.01	109,600 ± 917				N	7.11 ± 0.01	92,867 ± 643
**7**	N	6.60 ± 0.03	119,867 ± 306	N	6.77 ± 0.03	87,800 ± 200				N	7.09 ± 0.02	32,667 ± 306
**14**	N	6.58 ± 0.02	113,867 ± 115	N	6.80 ± 0.01	102,800 ± 600				N	7.09 ± 0.01	33,400 ± 200
**Accelerated Stability Testing (14 Days)—Temperature Cycles**												
**Before**	N	6.60 ± 0.32	161,000 ± 1217	N	6.56 ± 0.01	180,733 ± 22,689				N	6.55 ± 0.09	108,333 ± 416
**After**	N	7.21 ± 0.04	171,200 ± 20101	N	6.61 ± 0.03	156,533 ± 306				N	6.48 ± 0.01	38,467 ± 306
**Time (Days)**	**Gel**			**Gel + E**			**Gel + Etho**			**Gel + E-Etho**		
	**OC**	**pH**	**Viscosity**	**OC**	**pH**	**Viscosity**	**OC**	**pH**	**Viscosity**	**OC**	**pH**	**Viscosity**
**Accelerated Stability Testing (3 Months)—RC**												
**0**	N	8.88 ± 0.14	168,333 ± 306	N	6.34 ± 0.01	104,000 ± 200				N	6.77 ± 0.03	33,467 ± 306
**30**	N	8.79 ± 0.01	44,733 ± 416	N	6.09 ± 0.01	70,067 ± 611				N	6.62 ± 0.04	40,600 ± 200
**60**	N	8.77 ± 0.03	45,667 ± 757	N	6.20 ± 0.01	53,000 ± 1058				N	6.73 ± 0.04	36,533 ± 1332
**90**	N	8.80 ± 0.01	186,467 ± 8612	N	6.30 ± 0.01	126,000 ± 9035				N	6.90 ± 0.01	113,133 ± 1553
**Accelerated Stability Testing (3 Months)—RT**												
**0**	N	6.31 ± 0.06	194,533 ± 503	N	6.78 ± 0.02	167,200 ± 400				N	8.50 ± 0.01	21,800 ± 200
**30**	N	6.56 ± 0.03	42,933 ± 416	N	6.51 ± 0.01	50,533 ± 115				N	7.94 ± 0.01	97,800 ± 529
**60**	N	6.50 ±0.02	105,000 ± 6630	N	6.58 ± 0.04	109,133 ± 3775				N	7.44 ± 0.02	95,267 ± 8425
**90**	N	6.51 ± 0.01	132,600 ± 1929	N	6.67 ± 0.01	186,933 ± 2023				N	7.17 ± 0.03	56,400 ± 200
**Accelerated Stability Testing (3 Months)—AC**												
**0**	N	5.89 ± 0.04	159,267 ± 2444	N	7.42 ± 0.02	145,200 ± 200				N	8.02 ± 0.02	127,333 ± 503
**30**	N	6.33 ± 0.03	60,867 ± 416	N	6.87 ± 0.06	54,400 ± 200				N	7.52 ± 0.01	30,467 ± 833
**60**	N	6.36 ± 0.03	55,800 ± 2163	N	6.80 ± 0.01	72,467 ± 5623				N	7.37 ± 0.01	27,867 ± 702
**90**	N	6.47 ± 0.02	145,733 ± 1026	N	6.96 ± 0.02	168,733 ± 16,931				N	6.95 ± 0.04	100,600 ± 1562

**Table 3:** Skin compatibility test for Gel + E and Gel + E-Etho (*n* = 6 each group, mean ± S.D.).

Samples	Control Time after Patch Removal	Reactive Subjects (*n*)	Types of Reaction	Mean Daily Irritation Score (Mdis)
**Gel + E**	15 min	0	None	0 ± 0
	24 h	0	None	0 ± 0
	48 h	0	None	0 ± 0
**Gel + E-Etho**	15 min	0	None	0 ± 0
	24 h	0	None	0 ± 0
	48 h	0	None	0 ± 0

## Data Availability

The data presented in this study are available on request from the corresponding author.

## Supplementary Materials

The following are available online at https://www.mdpi.com/article/10.3390/ph14050467/s1, Figure S1. Chromatogram of methanolic elderflowers extract at 280 nm.
