# Supplementary material for: A Newfangled Collagenase Inhibitor Topical Formulation Based on Ethosomes with Sambucus nigra L. Extract
Source: Pharmaceuticals (Basel). 2021 May 15;14(5):467. doi: 10.3390/ph14050467 (PMC8155848; doi:10.3390/ph14050467)
Supplement: Supplementary file 1 [file pharmaceuticals-14-00467-s001.zip › pharmaceuticals-1200734-supplementary/Supplementary material_/supplementary material figure caption.pdf]

Figure S1- Chromatogram of methanolic elderflowers extract at 280 nm (1 – Malic Acid; 2 – Chlorogenic Acid; 3 – Luteolin-7-O-glucoside; 4 – Rutin; 5 – Quercetin-4-O-glucoside; 6 – Isoquercetin; 7 – Isorhamnetin-3-rutinoside; 8 – Isorhamnetin-3-O-glucoside; 9 – Eriodictyol; 10 – Naringenin). The MRM conditions were previously established with several standards belonging to an in-house library, enabling a rapid identification of metabolites in a single HPLC- MS/MS run.
