# Supplementary figures and images for: A Newfangled Collagenase Inhibitor Topical Formulation Based on Ethosomes with Sambucus nigra L. Extract
Source: Pharmaceuticals (Basel). 2021 May 15;14(5):467. doi: 10.3390/ph14050467 (PMC8155848; doi:10.3390/ph14050467)

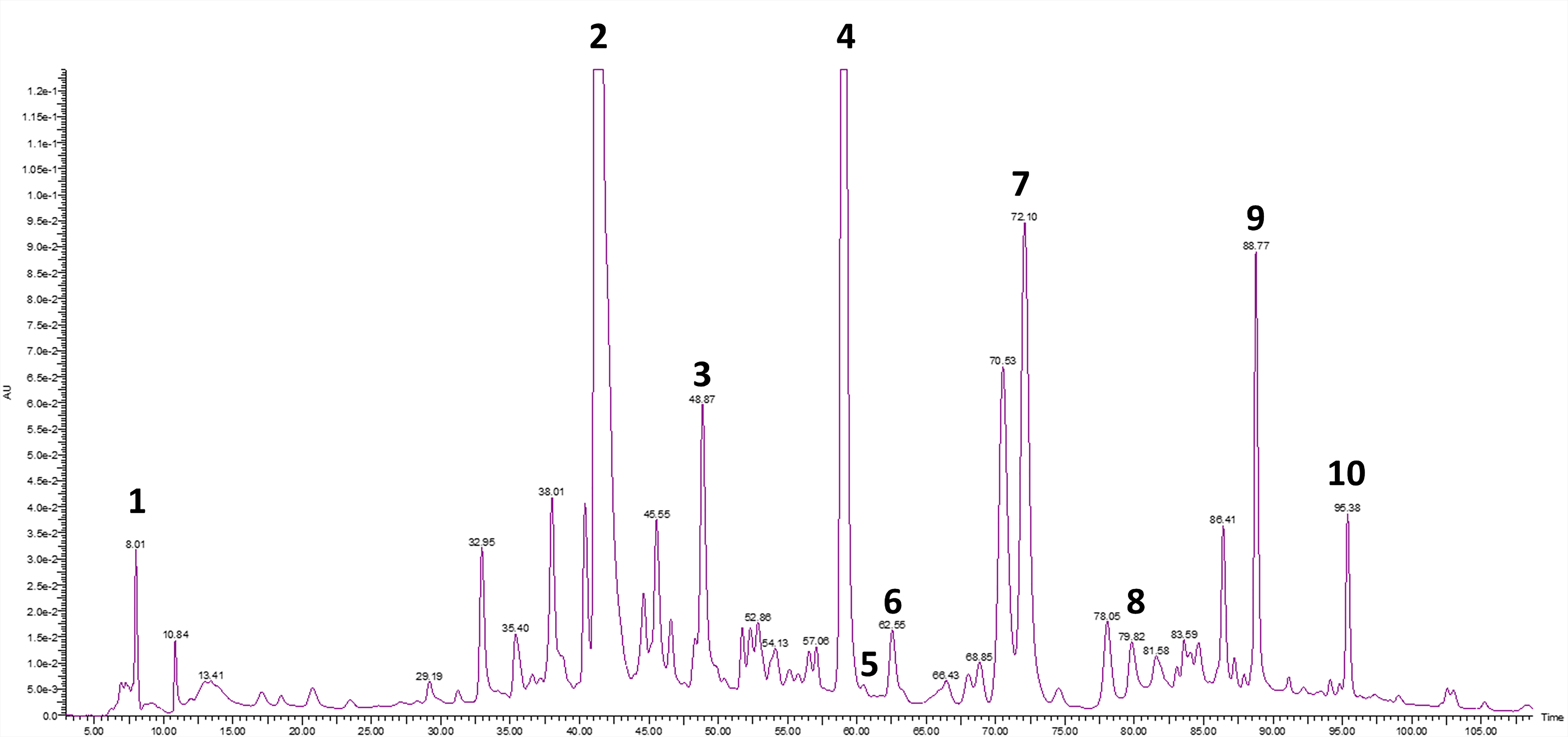

Supplement: Supplementary file 1 [file pharmaceuticals-14-00467-s001.zip › pharmaceuticals-1200734-supplementary/Supplementary material_/Supplementary Figure.tif]
